# Compaction Evolution and Mechanisms of Granular Materials Due to Gyratory Shearing

**DOI:** 10.3390/ma17225525

**Published:** 2024-11-12

**Authors:** Teng Man

**Affiliations:** 1 College of Civil Engineering, Zhejiang University of Technology, Hangzhou 310023, China; manteng0520@zjut.edu.cn; 2 Department of Civil, Environmental, and Geo-Engineering, University of Minnesota, Minneapolis, MN 55455, USA

**Keywords:** granular materials, compaction, gyratory shearing

## Abstract

Granular systems, no matter whether they are dry or saturated, are commonly encountered in both natural scenarios and engineering applications. In this work, we tackle the compaction problem of both dry and saturated granular systems under gyratory shearing compaction, where particles are subjected to constant pressure and continuous shear rate, which is quite different from the traditional cyclic shearing compaction. Such phenomena are crucial to the compaction of asphalt mixtures or soils in civil engineering and can be extended to other areas, such as powder processing and pharmaceutical engineering. In this study, we investigated the behavior of both dry and fully saturated mono-dispersed granular materials under gyratory shearing compaction using the discrete element method (DEM) and found that the gyratory speed or interstitial fluid viscosity has almost no impact on the compaction behavior, while the pressure and the particle size play more important roles. Additionally, it is the inertial time scale which dictates the compaction behavior under gyratory shearing in most cases; meanwhile, the viscous time scale can also have influence in some conditions. This work determines the similarity and unity between the granular gyratory compaction and the rheology of granular systems, which has direct relevance to various natural and engineering systems.

## 1. Introduction

Granular materials are frequently encountered and commonly used in nature and engineering practices such as mitigation of natural hazards, pharmaceutical engineering, food processing, civil engineering, etc. [1,2]. Granular materials in civil engineering are often subjected to complex loading conditions or loading path or have complex particle shapes. One kind of loading condition, which is common in powder processing and the manufacture of asphalt mixtures, is the compaction of granular materials (or granular–fluid systems). In most cases, we would like to have our concrete or asphalt pavement with as few air voids as possible. To achieve compaction under both pressure and continuous shearing, a so-called gyratory compactor is often used in the pavement industry to achieve denser asphalt mixtures. However, we know very little about the physics behind this kind of compaction when particles are subjected to continuous gyratory shearing (different from cyclic shearing, where particles dilate and relax due to the cyclic excitation, i.e., the dilation/relaxation cycle, and tapping compaction, where grains also collapse to a denser state because of dilation/relaxation cycles).

Most previous research on granular compaction has focused on mono-disperse spherical particle systems without interstitial fluids (dry granular material) under tapping, vibrating, or cyclic shearing. Mehta et al. [3,4] proposed a two-exponential equation with two relaxation time scales to describe the compaction behaviors based on experimental results [5].

(1) $$\begin{array}{c} \phi \left(t\right)=a_{0}-a_{1}\exp\left(-a_{3}t\right)-a_{4}\exp\left(-a_{5}t\right) \end{array}$$

where ϕ(t) is the solid fraction of granular materials at time *t*, and $a_{0}$, $a_{1}$, $a_{2}$, $a_{3}$, $a_{4}$, $a_{5}$ are fitting parameters obtained from experimental data. The two exponential terms can be associated with the independent and collective motion of particles during the compaction process. Knight et al. [5,6] proposed a logarithmic equation with the following form (Equation (2)) to better fit the compaction behavior of granular materials.

(2) $$\phi \left(t\right)=\phi_{f}-\frac{\Delta \phi_{\infty }}{1+B\ln\left(1+t/\tau \right)}$$

where $\phi_{f}$, $\Delta \phi_{\infty }$, *B*, and τ are fitting parameters. Meanwhile, the Renne group found that the compaction curve can also be fitted using a stretched exponential curve as follows [7,8,9]:

(3) $$\begin{array}{c} \phi \left(t\right)=\phi_{f}-\left(\phi_{f}-\phi_{0}\right)e^{-{\left(t/\tau \right)}^{\beta }} \end{array}$$

In all these instances, the evolution of granular systems into a denser configuration is partly attributable to the dilation–relaxation cycle that occurs during the loading process. For instance, when subjected to tapping excitation, particles initially expand and subsequently settle into their lowest-energy state during the relaxation phase. However, this phenomenon is absent in gyratory compaction, where particles are constantly subjected to a steady load. Consequently, it is challenging to predict the hidden time scales inherent to this problem, making it difficult to quantify the compaction behavior of the gyratory shearing process.

In this paper, the author begins by presenting the problem at hand, along with the boundary conditions of gyratory shearing compaction and its applications in asphalt mixture manufacturing. Subsequently, the paper will elaborate on computational simulations employing the discrete element method (DEM) [10,11], which will be used to conduct a basic parametric study on the gyratory compaction of mono-disperse granular assemblies comprising 5000 particles. Based on the simulation outcomes, the paper will discuss the impact of confining pressure, gyratory speed, and interstitial fluid viscosity and will further deduce insights into the nature of granular material compaction under gyratory conditions, offering perspectives on the time scales that govern compaction rates.

## 2. Simulation Setup

**Figure 1:**
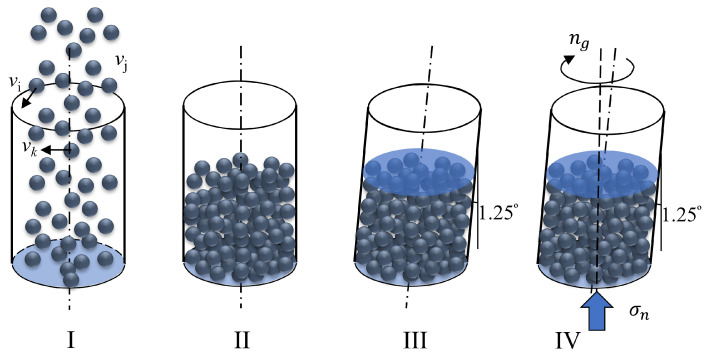
Configuration of the gyratory compactor and the model setup in the DEM simulation with four steps: (**I**,**II**) dropping particles, (**III**) adding the top plate and tilt the cylindrical ring, and (**IV**) starting the gyratory compaction.

Gyratory compaction is a compaction technique frequently employed in pavement engineering to attain a denser asphalt mixture in a laboratory. We note that a field compaction of asphalt mixture is quite different from a gyratory compaction in terms of the boundary conditions, but their main influential factors, i.e., pressure and shear, are similar [12]. Throughout the gyratory compaction process, the material undergoes consistent vertical pressure and gyratory movement, as depicted in Figure 1 and also illustrated in Man and Hill [13] and Man et al. [14]. The figure illustrates the setup of a gyratory compactor, which consists of particles placed within a cylindrical “ring” and confined by a stationary top plate and a movable bottom plate. While the top plate remains fixed in the vertical axis, the bottom plate ascends while exerting a constant pressure, denoted as $\sigma_{n}$. The cylindrical ring is subjected to a gyratory motion characterized by a speed of *n* (in SI units, revolutions per second or revolutions per minute) and a gyratory angle θ. This gyratory motion imparts an average shear rate to the granular system, which can be expressed as $\dot{\gamma }=2\pi \theta n$.

The configuration of DEM simulations parallels that of conventional asphalt mixture compaction experiments, as depicted in Figure 1 and also shown in the Supplementary Video. Initially, particles are randomly dispensed (Figure 1I) into a cylindrical container to create a random loose packing (Figure 1II). Subsequently, in the simulation, the cylindrical ring is slightly inclined to introduce a gyratory angle of θ, and a top plate is applied to the cylindrical container. The granular assemblies, initially loosely packed, are then subjected to loading and shearing under constant pressure and a steady gyratory speed (Figure 1IV). In summary, a gyratory compaction simulation of granular materials is the same as that happening in a SuperPave gyratory compaction test of hot-mixed asphalt. It has four steps: (1) we drop particles into the container; (2) we tilt the column to a certain angle; (3) we add a top plate to the system; and (4) we perform the gyratory compaction. We only need to perform a simulation of Steps (1–3) once to obtain the initial positions of all the discrete elements so that we can perform Step (4) with exactly the same initial condition. Also, we note that tilting the cylinder does not influence the results of the compaction since this step is embedded into the initial condition of the gyratory compaction.

Within our model, two categories of particle interactions are taken into account. We note that, in the discrete element method, each element can be considered as a particle; thus, the particle interaction is not limited to the interaction between spherical particles but also represents the interactions between spherical particles and boundary elements, where a boundary element is also a type of particle. The first type of interaction pertains to the physical contact between particles. Upon contact, the interaction forces between two particles are computed using the Hertz–Mindlin contact model [15,16], with the normal and tangential contact forces determined by the following equations:

(4a) $$\begin{array}{c} F_{c}^{\mathrm{ij},n}=-k_{n}\delta_{n}^{1.5}-\eta_{n}\delta_{n}^{0.25}{\dot{\delta }}_{n} \end{array}$$

(4b) $$\begin{array}{c} F_{c}^{\mathrm{ij},t}=\min\left(-k_{t}\delta_{n}^{0.5}\delta_{t}-\eta_{t}\delta_{n}^{0.25}{\dot{\delta }}_{t},\;\mu_{p}F_{c}^{\mathrm{ij},n}\right) \end{array}$$

where $F_{c}^{\mathrm{ij},n}$ and $F_{c}^{\mathrm{ij},t}$ are normal and tangential contact forces acting on particle *i* from particle *j*. $\delta_{n}$ is the overlap between particles in normal direction in DEM simulation, which is given by $\delta_{n}=R_{i}+R_{j}-\left|{\vec{r}}_{i}-{\vec{r}}_{j}\right|$, where $R_{i}$ and $R_{j}$ are particle radii, and ${\vec{r}}_{i}$ and ${\vec{r}}_{j}$ are position vectors of two particles. $\delta_{t}$ is the corresponding tangential deformation at the contact point between two particles, and $\mu_{p}$ is the coefficient of friction between particles. The same contact law applies to the interaction between boundaries and spherical particles. The second type of particle interaction is considered when the interstitial viscous fluid exists among particles, where the lubrication effect can be calculated accordingly [13,17,18,19].

(5a) $$\begin{array}{c} F_{v}^{\mathrm{ij},n}=6\pi \eta_{f}R_{\mathrm{eff}}^{2}\frac{{\dot{\delta }}_{g}}{\delta_{g}}\;, \end{array}$$

(5b) $$\begin{array}{c} F_{v}^{\mathrm{ij},t}=6\pi \eta_{f}R_{\mathrm{eff}}v_{t}^{\mathrm{rel}}\left[\frac{8}{15}\ln\frac{R_{\mathrm{eff}}}{\delta_{g}}+0.9588\right]\;, \end{array}$$

where $F_{v}^{\mathrm{ij},n}$ and $F_{v}^{\mathrm{ij},t}$ are normal and tangential lubrication forces between particle *i* and particle *j*. $\eta_{f}$ is the interstitial fluid viscosity and $R_{\mathrm{eff}}$ is the effective radius calculated based on the radius of two contacting particles. $v_{t}^{\mathrm{rel}}$ is the relative tangential velocity. $\delta_{g}$ is the gap between the nearest surface of two particles. The lubrication forces will diminish once the distance between particles exceeds the average particle diameters, $\delta_{\max}=\bar{d}$, and will remain invariant to distance once the particle distance is smaller than $\delta_{min}=0.1\bar{d}$, which represents the roughness of the particles. The same lubrication interaction is also applied to the interaction calculation between boundaries and spherical particles. No global drag force is considered in our simulation since we focus more on systems where the fluid can travel together with the particles. We assume that there is no relative motion between interstitial fluid and individual particles. Additionally, in this work, we focus on granular–fluid systems in fully saturated states which exceed the pendulum or capillary stage, so we do not need to consider the effect of capillary forces between particles (this is also similar to that of asphalt mixtures where viscous forces play a more important role). We note that the existence of capillary forces among particles is governed by the existence of solid–gas–liquid interfaces, which usually introduced liquid bridges between adjacent particles. In a fully saturated condition of granular materials, the lack of solid–gas–liquid interfaces leads to the neglect of any capillary effects [20].

The same as in other discrete element simulations, we can update the motion of each object of a system using Newton’s law based on the calculated forces acting on each element. We note that the contact forces between the boundary elements, i.e., top and bottom plates and the cylindrical ring, are also calculated in the same manner as shown in Equations (4) and (5). We determined the parameters for the particle interaction law using the same methodology as Hill and Tan [15]. The particles’ elastic modulus is 30 GPa, Poisson’s ratio is 0.2, and density is 2650 kg/m^3^ and the inter-particle friction coefficient is set to 0.2. Particle sizes are uniformly distributed between 0.8 and 1.2 times the mean diameter, $\bar{d}$, which is 2.0 mm in most cases. The cylindrical ring’s diameter is 20 times the average particle size. The gyratory angle, θ, is maintained at 0.25^∘^, consistent with asphalt mixture gyratory compaction practices. In our study, we varied the normal pressure, $\sigma_{n}$, from 10 kPa to 1000 kPa, the gyratory speed, $n_{g}$, from 3 rpm to 100 rpm, and the interstitial fluid viscosity, $\eta_{f}$, from 0 cP (no fluid) to 1000 cP (equivalent to glycerin). Subsequently, we also explored the impact of particle size to reinforce our analysis of the dominant time scale in the gyratory shearing compaction of granular systems.

## 3. Results and Discussions

### 3.1. Behavior of Granular Materials During the Gyratory Shearing Compaction

**Figure 2:**
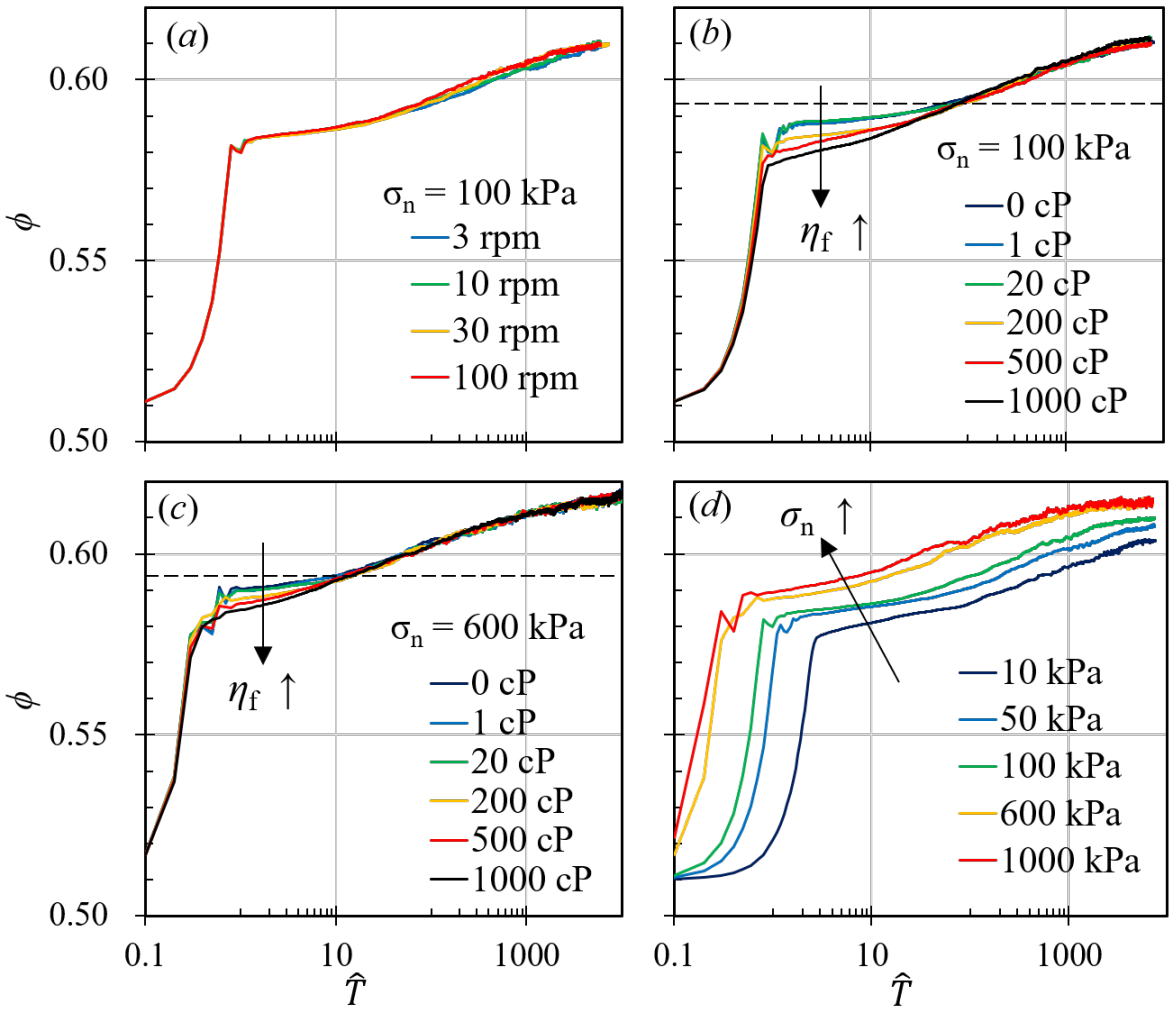
Compaction curves of granular materials with different conditions. In (**a**), we plotted the results of simulations with different gyratory speeds while keeping the pressure and the interstitial fluid viscosity constant at 100 kPa and 100 cP, respectively. We plotted the simulations with different interstitial fluid viscosity in (**b**,**c**). However, the pressure is different in these two sets of simulations. In (**d**), we have the relationship between solid fraction and dimensionless time, $\hat{T}$, of simulations with different pressures.

Figure 2 shows us the general behavior of granular materials during gyratory shearing compactions. Before compaction happens, the granular assembly is in a loosely packed state, where the initial packing fraction is around 0.51 and the initial porosity is approximately 0.49. After the gyratory compaction starts, Figure 2a shows that the gyratory compaction of granular materials can be roughly classified into two stages: (i) At the beginning of the compaction, the solid fraction increases rapidly from a loosely, randomly packed solid fraction to a randomly packed fraction, and we can witness a swift decrease in the porosity of the granular system. (ii) Afterwards, the solid fraction exceeds the randomly packed fraction and has logarithmic growth with respect to time. Between these two stages, there is a seemingly intermediate stage where the slope of the curve is slightly smaller than that in the logarithmic growth region. However, the second stage can be further divided into sub-stages when we change other variables, such as the viscosity of the interstitial fluid.

The detailed explanations of the $\phi -\hat{T}$ relationships with respect to different gyratory speeds, different fluid viscosities, and different pressures will be presented in the following sections. However, we ought to explain the way we normalize the parameters. The solid fraction, ϕ, is dimensionless, but the compaction time, *t*, is dimensional. Without knowing the characteristic time scale associated with the gyratory compaction scenario, we initially non-dimensionalize *t* with a time factor of $\sqrt{{\bar{r}}_{p}/g}$, where *g* is the gravitational acceleration and ${\bar{r}}_{p}$ is the average particle diameter. In this way, the dimensionless time becomes

(6) $$\begin{array}{c} \hat{T}\equiv t/\sqrt{{\bar{r}}_{p}/g}=t\sqrt{g/{\bar{r}}_{p}}\;. \end{array}$$

**Figure 3:**
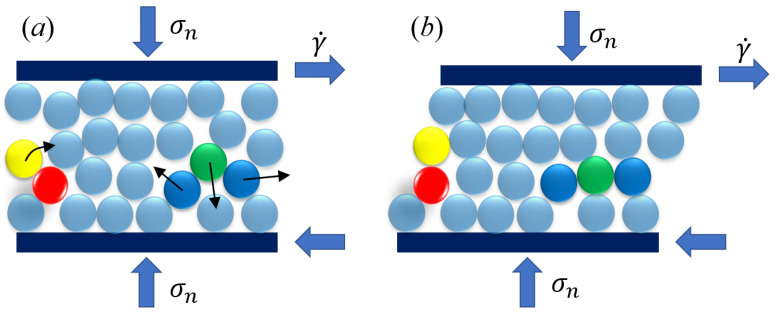
Schematic plot of both the shearing dilation and the compressive relaxation. (**a**) shows the start of a shearing motion and (**b**) shows the final state after a shear step.

Based on the general behavior of the $\phi -\hat{T}$ relationships, in the gyratory shearing compaction of a granular system, particles may undergo two distinct types of motion, as illustrated in Figure 3. Initially, particles experience shearing, characterized by the relative displacement of one layer over another. The temporal scale of this shearing motion is inversely proportional to the shear rate, $\dot{\gamma }$, and can be expressed as $1/\dot{\gamma }$. Secondly, particles are subject to a relaxation effect, where the application of confining pressure facilitates the compression of particles from one layer into another, thereby increasing the density of the granular assembly. The temporal scale of this relaxation process is related to the grain diameter, *d*; the confining pressure, $\sigma_{n}$; and the particle density, $\rho_{p}$ and can be quantified as $\bar{d}/\sqrt{\sigma_{n}/\rho_{p}}$, where $\bar{d}$ is the mean particle diameter. When an interstitial fluid is present, and its viscosity, $\eta_{f}$, governs the behavior of the granular–fluid system, the temporal scale of motion can be represented as $\eta_{f}/\sigma_{n}$. Figure 3 provides a detailed depiction of the particle motions associated with these two types of granular behavior. Under the influence of both normal stress, $\sigma_{n}$, and shear rate, $\dot{\gamma }$, particles exhibit complex interactions. For instance, the shearing effect causes a yellow particle to slide over a red particle in a sub-layer, increasing its height and contributing to the dilation of the system. Conversely, a green particle is compressed to fill the void between two blue particles, promoting a denser state of the system. During gyratory shearing compaction, these two types of particle motions counterbalance each other, ultimately leading to a condensed state of the granular assembly. We note that, although Figure 3 is shown in a two-dimensional manner, the gyratory compaction is always three-dimensional, as shown in Figure 1.

### 3.2. Influence of Gyratory Speed and the Viscosity of Interstitial Fluid

Figure 2a illustrates the compaction curves for granular materials subjected to various gyratory speeds while maintaining a constant pressure of 100 kPa and interstitial fluid viscosity of 100 cP. The curves delineate the relationship between the solid fraction, ϕ, and the normalized time, $\hat{T}=t\sqrt{g/{\bar{r}}_{p}}$, as introduced in the previous section, where ${\bar{r}}_{p}$ represents the average particle radius, calculated as ${\bar{r}}_{p}=0.5\bar{d}$, and $\bar{d}$ is the average particle diameter. Previous studies have indicated that cyclic shearing significantly affects the compaction behavior of granular systems. In contrast, our research, as depicted in Figure 2a, demonstrates that alterations in gyratory speed, which correspond to changes in the average shear rate, do not substantially influence the overall compaction dynamics. Figure 2a shows that, when we keep the compaction pressure and the interstitial fluid viscosity constant, changing the gyratory speed alone has no effects on the $\phi -\hat{T}$ curve. All four curves shown in Figure 2a have exactly the same behavior: at the beginning of the compaction, the granular systems go through the same rapid compaction period, where the solid fraction grows rapidly from approximately 0.51 to approximately 0.56, after which, systems with different gyratory speeds experience the same slow compaction period, as the solid fraction increase from 0.58 to almost 0.61. In other word, the porosities of systems with different gyratory speeds decrease from 0.49 to 0.42 and then to 0.39 in exactly the same manner.

**Figure 4:**
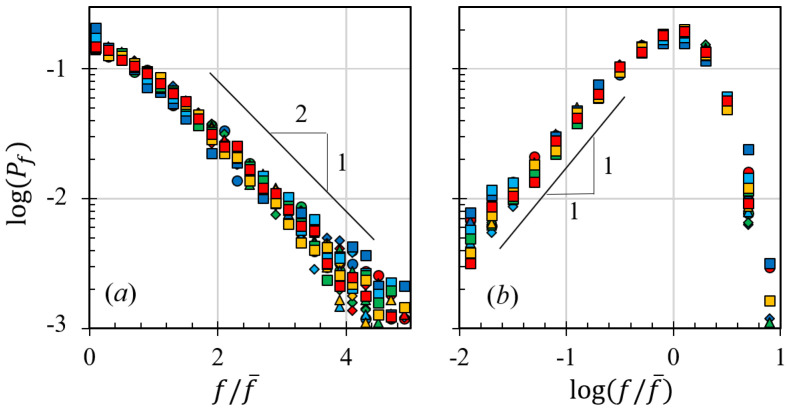
Histogram of the contact forces between particles: (**a**) the histogram in a linear-log coordinate system and (**b**) the histogram in a log-log coordinate system. Markers 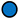, 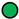, 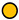, 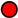 represent simulation results of different gyratory speeds. 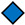, 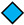, 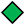, 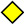, 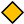, 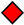 represent simulation results of different viscosity when $\sigma_{n}=100$ kPa, while 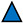, 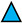, 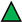, 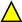, 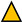, 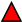 represent simulation results of different viscosity when $\sigma_{n}=600$ kPa. 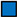, 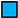, 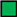, 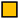, 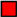 represent simulation with different pressures.

Furthermore, the gyratory speed appears to have no significant effect on the statistical behavior of the granular materials, as shown in Figure 4. In Figure 4, we plot the histograms of the ratio between the contact forces among particles, *f*, and the mean contact force, $\bar{f}$, where the circles with different colors represent the simulation results of systems with different gyratory speeds. As we can see from this figure, the probability density function of $f/\bar{f}$ is insensitive to the change of gyratory speeds.The figure reveals that the distribution of normalized contact forces exhibits an exponential decay for forces *f* exceeding the mean contact force $\bar{f}$ (shown in the right figure) and a power-law tail for forces below the mean (shown in the left figure). We can conclude that the functional form of the force probability distribution in a gyratory shearing compaction of a granular system is the same as that in a sand pile, where $P_{f}\left(f/\bar{f}\right)∼{\left(f/\bar{f}\right)}^{\alpha_{f}}$ when $f/\bar{f}<1.0$, and $P_{f}\left(f/\bar{f}\right)∼\exp-\beta_{f}f/\bar{f}$ when $f/\bar{f}>1.0$. $\alpha_{f}$ and $\beta_{f}$ seem to be affected by the compaction pressure, which will be discussed in Section 3.3.

We also examine the impact of interstitial fluid viscosity, presenting results from two sets of simulations in Figure 2b,c. In one set (Figure 2b), we hold the pressure and gyratory speed constant at 100 kPa and 30 rpm and vary the viscosity from 0 cP to 1000 cP. Although one might intuitively expect a significant influence of interstitial fluid viscosity on compaction behavior, as suggested by Fiscina et al. [21], our findings indicate that the presence of interstitial fluid primarily affects the intermediate stages of gyratory compaction. Figure 2b shows the relationship between ϕ and $\hat{T}$ for systems with different interstitial fluid viscosities, $\eta_{f}$. It shows that when $\eta_{f}$ is large, the system will directly transition from the rapid compaction phase to the logarithmic growth phase. However, decreasing $\eta_{f}$ results in a larger solid fraction at the end of the rapid compaction phase while introducing a plateau of the solid fraction. For example, at the end of the rapid compaction stage, the system with $\eta_{f}=1000$ cP has a solid fraction of roughly 0.575 (or porosity of 0.425), while the system with $\eta_{f}=0$ cP reaches a solid fraction of approximately 0.59. This indicates that the viscosity of the interstitial fluid may hinder the compaction process, but, eventually, after the plateau, the influence of $\eta_{f}$ will diminish.

Similarly in Figure 2c, we plot the relationship between the solid fraction, ϕ, and the dimensionless time, $\hat{T}$, when the pressure is 600 kPa. Compared to Figure 2b, the time period where the interstitial fluid has influences (the period of the solid fraction plateau, which can be influenced by $\eta_{f}$ decreases as we increase the confining pressure. In this case, the pressure is much higher (600 kPa, compared to 100 kPa in Figure 2b), which makes the granular assembly to reach a certain solid fraction much earlier than that in Figure 2b. Interestingly, the solid fraction which marks the end of interstitial fluid interference is the same for different pressure conditions. When the solid fraction is larger than ≈0.59, the viscosity of the interstitial fluid has almost no effect on the gyratory compaction behavior of the granular materials, no matter how large the viscosity becomes. Meanwhile, this dividing point for the solid fraction happens to be equal to the loose, randomly packed fraction [22]. Also, Man and Hill [13,23] found that, in a granular–fluid system, the frictional rheology (the $\mu_{\mathrm{eff}}-\phi$ relationship) followed two pathways depending on the magnitude of the viscous number $I_{v}=\eta_{f}\dot{\gamma }/\sigma_{n}$ in a shearing system, but these two different pathways merged into one curve at approximately ϕ=0.59 where fluid viscosity can no longer influence the rheology of the granular materials. Our results in the gyratory compaction also conclude with a 0.59 solid fraction terminating the influence of the interstitial fluids.

Based on the influence of the interstitial fluid viscosity, we can classify the whole gyratory compaction process into three stages: (i) the fast compaction stage, where the solid fraction of the granular system grows rapidly and is unaffected by the change of the fluid viscosity; (ii) the intermediate stage, which is signatured by the obvious influence of fluid viscosities and during which the system solid fraction is usually below 59.5%; (iii) the creeping stage, where the system is compacted in a logarithmic manner until it reaches the jamming solid fraction, beyond which we regard as out of the scope of this work. Meanwhile, similar to the influence of the gyratory speed, changing the viscosity of the interstitial fluid results in negligible influences on the histogram of $f/\bar{f}$. This indicates that, in a fully saturated condition and while the system is under gyratory shearing, changing the fluid viscosity does not necessarily alter the contact network.

### 3.3. The Pressure Influence and the Dominating Time Scale

In contrast to the effects of gyratory speed and interstitial fluid viscosity, altering the pressure on granular packing substantially modifies the compaction behavior. Higher pressures allow granular materials to attain a significantly higher solid fraction and reach a specific solid fraction more swiftly than simulations conducted at lower pressures, as depicted in Figure 2d. Despite these variations in compaction behavior across different pressures, the statistics of inter-particle forces remain largely unchanged (as illustrated in Figure 4). If we examine this figure more carefully, we can find that, in the right figure of Figure 4 and when $f/\bar{f}<0.1$, systems with lower pressures exhibit higher probability of establishing weak-force networks. This subtle difference may result in different fluctuation behaviors for systems with different pressure. Since larger fluctuations often lead to smaller solid fractions, smaller compaction pressure inevitably results in smaller ϕ. This observation prompts the question of what drives these alterations in compaction behavior, which our study seeks to answer. Previous research frequently correlates the compaction curve with time scales yet often fails to explore the details or the influencing factors. In our study, we regard the compaction behavior as a consequence of the interplay between the shearing time scale, $\tau_{s}=1/\dot{\gamma }$; the viscous time scale, $\tau_{v}=\eta_{f}/\sigma_{n}$; and the inertial time scale, $\tau_{i}=d/\sqrt{\sigma_{n}/\rho_{p}}$, in accordance with the frictional rheology of granular materials [13,24,25].

**Figure 5:**
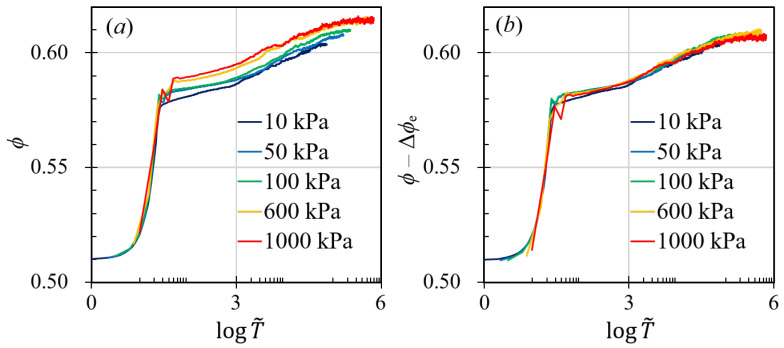
(**a**) Relationship between the solid fraction, ϕ, and the dimensionless time, $\tilde{T}=t\sqrt{\sigma_{n}/\rho_{p}}/\bar{d}$. (**b**) Relationship between the “visco-plastic” solid fraction, $\phi -\Delta \phi_{e}$, and the dimensionless time, $\tilde{T}$.

Upon examining Figure 2a–c, we can deduce that the impact of interstitial fluid viscosity and gyratory speed is negligible when compared to the inertial effect. Consequently, the compaction curve should be predominantly governed by the inertial time scale, $\tau_{i}=d/\sqrt{\sigma_{n}/\rho_{p}}$. By graphing ϕ against the normalized time $\tilde{T}=t/\tau_{i}=t\sqrt{\sigma_{n}/\rho_{p}}/\bar{d}$, we would expect the compaction curves to converge onto a single trajectory. However, as evidenced in Figure 5a, the curves diverge post-logarithmic stage. Intriguingly, at identical normalized times $\tilde{T}$, the slope of the curves remains roughly consistent across various pressure conditions, suggesting that an additional factor is absent from our current analysis.

Thus, we divided the compaction into “elastic” compaction and “visco-plastic” compaction. The “elastic” compaction can be calculated based on the solid fraction change of granular packing with the basic hexagonal close packing (HCP) given pressure condition $\sigma_{n}$. In this case, the forces between adjacent particles are $F_{c}=0.25\sigma_{n}\pi d^{2}=k_{n}\delta_{n}^{1.5}$, where $k_{n}=1.33\sqrt{R_{\mathrm{eff}}}E_{\mathrm{eff}}$ according to the Hertz contact model [15]. Based on this, we can calculate the elastic deformation $\delta_{n}$. For a monodispersed granular system, the effective radius $R_{\mathrm{eff}}$ is the same as the particle radius $r_{p}$ and the effective elastic modulus $E_{\mathrm{eff}}$ is the same as the particle elastic modulus $E_{s}$. Then, we can rewrite the overlap ratio $\delta_{n}/r_{p}$ as a function of the loading condition in a hexagonal close-packing condition as follows:

(7) $$\begin{array}{c} \frac{\delta_{n}}{r_{p}}={\left(\frac{3}{4}\pi \frac{\sigma_{n}}{E_{s}}\right)}^{2/3}\;. \end{array}$$

**Figure 6:**
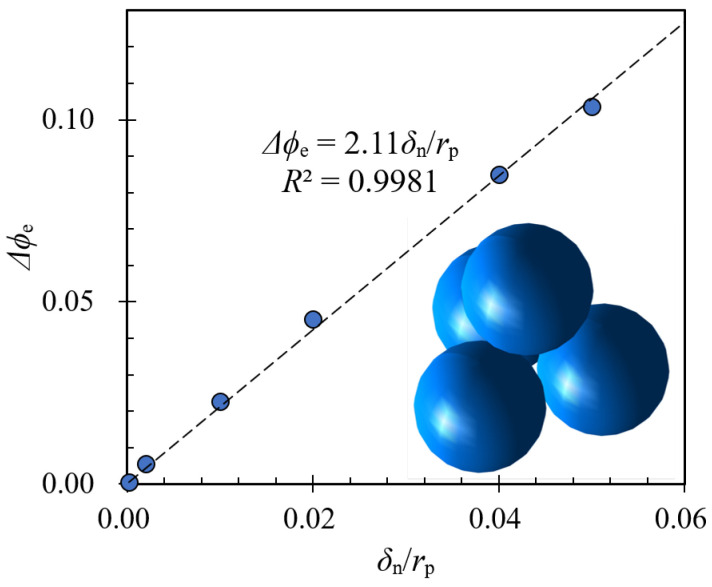
Relationship between the change in solid fraction, $\Delta \phi_{e}$, and the relative deformation between adjacent particles, $\delta_{n}/r_{p}$, where $r_{p}$ is the particle radius. The inset shows the basic structure of closely packed particles used for calculating the change in solid fractions.

Using a Monte Carlo algorithm, we could measure the approximate solid fraction given a contact overlap and calculate the relationship between $\delta_{n}$ and the “elastic” change of the solid fraction and plot the results in Figure 6, which shows that the elastic contribution of the solid fraction changes scales linearly with respect to the overlap ratio $\delta_{n}/r_{p}$. With these analyses, we could link the pressure $\sigma_{n}$ to the change in solid fraction $\Delta \phi_{e}$. In Figure 5b, we plotted the relationship between $\phi -\Delta \phi_{e}$ and $\tilde{T}=t\sqrt{\sigma_{n}/\rho_{p}}/\bar{d}$, which shows a good collapse of data among simulations with different pressures. This indicates that the compacted solid fraction of granular systems under gyratory compaction can be written as a combined function of both $\tilde{T}$ and $\Delta \phi_{e}$ with the following equation:

(8) $$\begin{array}{c} \phi \left(\sigma_{n},t\right)=F_{c}\left(t\frac{\sqrt{\sigma_{n}/\rho_{p}}}{\bar{d}}\right)+\xi_{e}\cdot {\left(\frac{3}{4}\pi \frac{\sigma_{n}}{E_{s}}\right)}^{2/3}\;, \end{array}$$

where $F_{c}\left(\cdot \right)$ is the function shown in Figure 5b, and $\xi_{e}\approx 2.11$ is a fitting parameter that comes from the relationship between $\Delta \phi_{e}$ and $\delta_{n}/r_{p}$. The influence of compaction pressure also stems from the force probability distribution presented in Figure 4, where changing $\sigma_{n}$ affects the extreme statistics of $f/\bar{f}$. When $f/\bar{f}>3$, simulations with smaller pressure have larger chances to experience relatively large contact force. Similarly, when $f/\bar{f}<0.1$, simulations with smaller pressure are also more likely to have relatively small contacts. These indicate that low compaction pressure leads to relatively large fluctuations, which may result in a smaller compaction solid fraction.

### 3.4. Influence of Particle Sizes

We have found that the compaction pressure plays a more important role than the viscosity of the interstitial fluid and the gyratory speed, and the time scale, $\tau_{i}=d/\sqrt{\sigma_{n}/\rho_{p}}$, is the key scaling factor for the gyratory compaction of granular systems. However, we should also investigate the influence the particle size, $r_{p}$, as this factor also influences the time scale $\tau_{i}=d/\sqrt{\sigma_{n}/\rho_{p}}$. In this case, we choose three different particle sizes ($r_{p}=$ 0.2 cm, 1.0 cm, and 5.0 cm) for our investigation. As we change the particle size, we also change the system size accordingly to ensure that the ratio between the system radius and the particle radius is kept constant. To eliminate the effect of the interstitial fluid, we set the fluid viscosity to 0.

**Figure 7:**
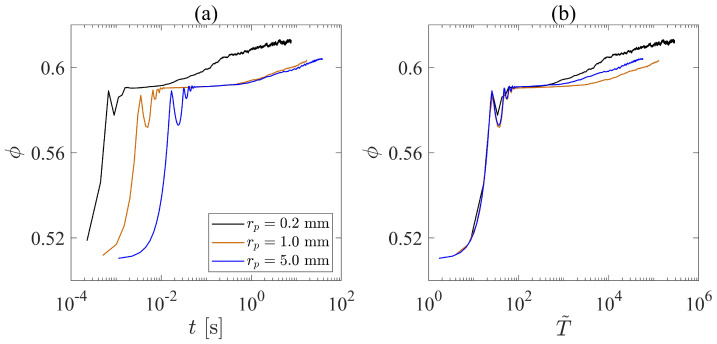
(**a**) The relationship between the solid fraction and time for systems with different particle sizes. (**b**) We vary the *x*-axis of Figure (**a**) to the dimensionless time $\tilde{T}=t/\left(d/\sqrt{\sigma_{n}/\rho_{p}}\right)$.

In Figure 7a, we plot the solid fraction against the real compaction time. We find that, during the rapid compaction stage, three different cases behave similarly. However, the granular system with smaller particle sizes reaches the intermediate stage earlier than the granular system with larger particles, which indicates that it is easier to compact a small-particle granular system than to compact a large-particle granular system. Additionally, the system with $r_{p}=0.2$ mm reaches the creeping stage much earlier than the other two cases. As we normalize the compaction time *t* with the inertial time scale $\tau_{i}=d/\sqrt{\sigma_{n}/\rho_{p}}$ and plot the results in Figure 7b, the transition points between the rapid compaction stage and the intermediate stage are unified into one single point, which shows that the transition between these two stages is dominated by the inertial time scale. However, the transition point between the intermediate stage and the creeping stage varies from case to case. Figure 7b shows that the system with $r_{p}=0.2$ mm reaches the creeping stage earlier, but the system with $r_{p}=5.0$ mm comes second, while the granular system with $r_{p}=1$ mm becomes the latest one among the three to reach the creeping stage in terms of the dimensionless time, $\tilde{T}=t/\left(d/\sqrt{\sigma_{n}/\rho_{p}}\right)$. The influence of particle sizes on the transition from the intermediate stage to the creeping stage is apparently complex and non-monotonic. We note that as we change the particle size, we also vary the system size accordingly. Thus, the geometric constraints for the three systems with different $r_{p}$ are exactly the same. Since we also keep the material’s Young’s modulus constant, the effective stiffness of the particles varies with the particle size, which may also contribute to the complex behavior of the size-induced compaction difference.

**Figure 8:**
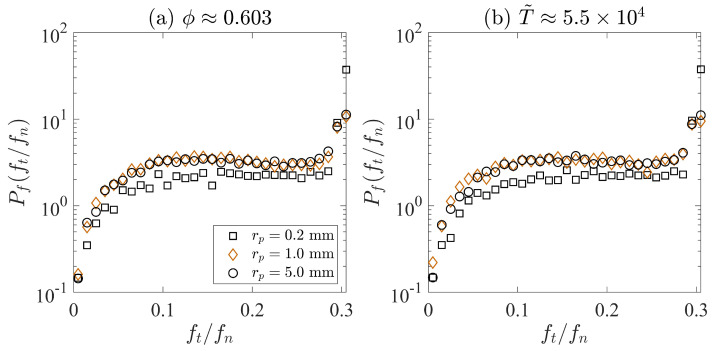
(**a**) Histogram of the ratio between the tangential and the normal contact forces, $f_{t}/f_{n}$, when three systems have the same compacted solid fraction. (**b**) Histogram of $f_{t}/f_{n}$, when three systems are the the same dimensionless time, $\tilde{T}$.

In addition to the influence of the effective stiffness, we argue that the size effect may also be due to the differences in frictional contacts among particles. Systems with more sliding contact lead to easier particle rearrangement, which results in larger solid fractions. Thus, we plot the histogram of the ratio between tangential and normal contact force, $f_{t}/f_{n}$, in Figure 8. Since we have set the contact frictional coefficient to be 0.3, particles have sliding contact when their $f_{t}/f_{n}$ values are approximately equal to 0.3. Two sets of simulation results are examined in this section. In Figure 8a, we focus on the histograms when three different cases are at the same compacted solid fraction (ϕ≈ 0.603), whereas, in Figure 8, we investigate the histograms when three different simulations are at the same dimensionless time, where $\tilde{T}\approx 5.5\times 10^{4}$. For Figure 8a,b, the system with $r_{p}=0.2$ mm has a larger probability to have $f_{t}/f_{n}\approx$ 0.3. This indicates that it is easier to find sliding contacts when the particle radius is 0.2 mm. The systems with $r_{p}=1.0$ mm and $r_{p}=5.0$ mm have larger probability values for $f_{t}/f_{n}<0.3$ than the system with $r_{p}=5.0$ mm, but the difference between the simulations with $r_{p}=1.0$ mm and $r_{p}=5.0$ mm is not obvious. Figure 8 shows that the system with $r_{p}=5.0$ mm has a slightly larger chance to have sliding contact, which may help improve the compactibility of the system. Detailed investigations on the contact network and particle stiffness are still needed in our future studies.

## 4. Further Discussions on the Reversibility and Irreversibility

**Figure 9:**
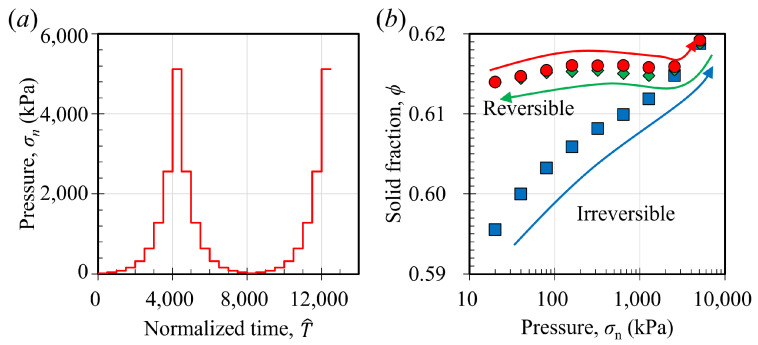
(**a**) shows the loading condition, where the compaction pressure varies between 20 kPa and 5120 kPa. (**b**) shows the relationship between compaction pressure and the steady-state solid fractions. Marker (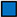) shows the cases of the change in solid fractions when we increase the pressure from 20 kPa to around 5120 kPa (the first loading process). Marker (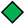) shows the results when we decrease the pressure from 5120 kPa to 20 kPa (the unloading process). Marker (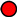) shows the results when we increase the pressure from 20 kPa to 5120 kPa again (the second loading process).

Next, we delve into the impact of confining pressure by alternating it between 20 kPa and 5120 kPa (refer to Figure 9a). Upon adjusting to a new pressure level, we maintain the system at this pressure for a duration of $\hat{T}=500$ to allow the system to achieve a steady-state compaction phase. We selected this particular loading sequence solely to examine the compaction dynamics when the pressure is incrementally increased and subsequently decreased. Observing the progression of the solid fraction in response to abrupt pressure changes is intriguing. In this simulation, we initiated the compaction of granular–fluid systems at a gyratory speed of 30 rpm and with an interstitial fluid viscosity of 200 cP. The initial confining pressure was set at 20 kPa. Upon achieving a steady-state compaction phase, which was maintained for a duration of 100 dimensionless time units, $\hat{T}=t\sqrt{g/{\bar{r}}_{p}}$, we augmented the compaction pressure by 100%. This procedure was iterated until the pressure attained a value of 5120 kPa (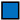), at which point we began a gradual unloading process, decrementing the pressure back to 20 kPa (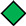). Following the unloading phase, we subjected the system to an additional loading cycle, increasing the pressure from 20 kPa to 5120 kPa (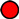) again. We note that the increase in pressure is not in a linear manner but in a logarithmic manner.

Figure 9b illustrates the correlation between the solid fraction and the compaction pressure. In the initial loading phase, as the pressure is elevated from 20 kPa to 5120 kPa, the steady-state solid fraction rises from roughly 0.595 to nearly 0.62. If we define the logarithmic compaction rate with respect to pressure as the slope of $\phi -\sigma_{n}$ curve in a log-linear coordinate system, $d\phi /d(\log\sigma_{n})$, the logarithmic compaction rate decreases with the increase of pressure when the solid fraction is approximately less than 0.61. When ϕ>0.61, $d\phi /d(\log\sigma_{n})$ increases and then keeps almost constant when we further increase the pressure. During the unloading phase, the solid fraction with respect to pressure initially tracks the same trajectory as the initial loading path until it reaches approximately 0.616 (denoted by the green arrow in Figure 9b). Below this solid fraction point, ϕ becomes largely independent of the confining pressure variations. Subsequently, in the second loading phase (denoted by the red arrow in Figure 9b), the solid fraction’s relationship with the confining pressure mirrors that of the unloading phase. Consequently, there are two distinct branches in the relationship between the confining pressure and the solid fraction, with the initial loading path being irreversible and the unloading phase along with the subsequent reloading being reversible. This behavior indicates that granular material can achieve random close packing even under relatively low-pressure conditions, a phenomenon akin to that observed in the tapping or shaking compaction of granular materials. The irreversibility of granular systems under gyratory compaction can be further linked to the statistical mechanics of granular matter; thus, we are further conducting related experimental and simulation works and plan to report them in future publications.

## 5. Conclusions

In the present study, we conducted a comprehensive analysis of the compaction dynamics of granular materials subjected to gyratory shearing based on the discrete element method with monodispersed spherical particles, where the numerical method is characterized by Hertz–Mindlin contact laws and lubrication theories. This compaction mechanism, which is a three-dimensional continuous shearing with a constant pressure, is distinct from those observed in traditional tapping or cyclic shearing processes as shown in Section 2 and the Supplementary Video. Our findings indicate that the gyratory speed of the gyratory shearing compaction has a negligible effect on the compaction behavior (Section 3.2). Also, we determined that the viscosity of the interstitial fluid exerts a secondary influence on compaction during the intermediate phase, while the particle size distribution predominantly affects the creeping phase of gyratory compaction as shown in Section 3.2 and Section 3.4. Notably, the applied pressure emerges as a primary determinant of the compaction outcome. Throughout the compaction process, three distinct time scales—the shearing time scale ($1/\dot{\gamma }$), the viscous time scale ($\eta_{f}/\sigma_{n}$), and the inertial time scale ($d/\sqrt{\sigma_{n}/\rho_{p}}$)—compete for dominance, facilitating the identification of the critical time scale pertinent to gyratory shearing compaction.

Furthermore, to ascertain the compaction solid fraction relative to dimensionless times, we have delineated the deformation of granular materials during compaction into two distinct phases: (i) “elastic” compaction and (ii) “visco-plastic” compaction. The “visco-plastic” compaction deformation, denoted as ($\phi -\Delta \phi_{e}$), is predominantly governed by the inertial time scale, $d/\sqrt{\sigma_{n}/\rho_{p}}$, as depicted in Figure 5b. Additionally, our investigation into the size effect of gyratory compaction of granular systems has revealed that smaller particles generally yield a higher probability of sliding contact, which in turn leads to increased solid fractions. We note that, as we change the particle size of a granular system, the size of the granular assembly is kept constant so that the size ratio between the system and the particle is unchanged. We have also examined the reversibility and irreversibility of gyratory compaction, demonstrating the complexity of this process and indicating the necessity for further research in subsequent studies. This research is particularly pertinent to the civil engineering sector, where the determination of compaction intensity and duration is crucial for maximizing economic benefits. Even though the analyses of this work is framed based on the quantitative and qualitative investigations of the compacted solid fraction, it can be easily transformed into porosity of the granular system, which is more acceptable to the civil engineering community. Moreover, such experiments could prove advantageous for future research endeavors aimed at understanding force chains in three-dimensional scenarios, potentially incorporating computed tomography technology.

## Data Availability

The raw data supporting the conclusions of this article will be made available by the authors on request.

## Supplementary Materials

The following are available at https://www.mdpi.com/article/10.3390/ma17225525/s1.
